# Use of dynamic contrast-enhanced MRI to measure subtle blood–brain barrier abnormalities^☆^

**DOI:** 10.1016/j.mri.2010.09.002

**Published:** 2011-04

**Authors:** Paul A. Armitage, Andrew J. Farrall, Trevor K. Carpenter, Fergus N. Doubal, Joanna M. Wardlaw

**Affiliations:** Clinical Neurosciences, School of Molecular and Clinical Medicine, Western General Hospital, University of Edinburgh, Edinburgh, EH4 2XU, Scotland, UK

**Keywords:** Blood–brain barrier, MRI, Permeability, Stroke

## Abstract

There is growing interest in investigating the role of subtle changes in blood–brain barrier (BBB) function in common neurological disorders and the possible use of imaging techniques to assess these abnormalities. Some studies have used dynamic contrast-enhanced MR imaging (DCE-MRI) and these have demonstrated much smaller signal changes than obtained from more traditional applications of the technique, such as in intracranial tumors and multiple sclerosis. In this work, preliminary results are presented from a DCE-MRI study of patients with mild stroke classified according to the extent of visible underlying white matter abnormalities. These data are used to estimate typical signal enhancement profiles in different tissue types and by degrees of white matter abnormality. The effect of scanner noise, drift and different intrinsic tissue properties on signal enhancement data is also investigated and the likely implications for interpreting the enhancement profiles are discussed. No significant differences in average signal enhancement or contrast agent concentration were observed between patients with different degrees of white matter abnormality, although there was a trend towards greater signal enhancement with more abnormal white matter. Furthermore, the results suggest that many of the factors considered introduce uncertainty of a similar magnitude to expected effect sizes, making it unclear whether differences in signal enhancement are truly reflective of an underlying BBB abnormality or due to an unrelated effect. As the ultimate aim is to achieve a reliable quantification of BBB function in subtle disorders, this study highlights the factors which may influence signal enhancement and suggests that further work is required to address the challenging problems of quantifying contrast agent concentration in healthy and diseased living human tissue and of establishing a suitable model to enable quantification of relevant physiological parameters. Meanwhile, it is essential that future studies use an appropriate control group to minimize these influences.

## 1 Introduction

The blood–brain barrier (BBB) is commonly studied using dynamic contrast-enhanced MRI (DCE-MRI) in diseases such as brain tumors [1], [2], [3] and multiple sclerosis [4], [5], [6] where a relatively large focally abnormal BBB is observed. There is increasing interest in using this imaging technique to identify more subtle BBB abnormalities, such as those which occur with normal ageing [7], dementia [7], [8], [9], [10], [11], [12], Alzheimer's disease [13], type II diabetes [14], cerebral microvascular disease [7], [15] and in nonenhancing multiple sclerosis lesions [16], [17]. These initial results suggest that DCE-MRI of subtle BBB disorders may provide useful information. However, maximum post-contrast signal differences are small, typically about 5% in gray matter and 1–2% in white matter, with changes over the imaging period being on the order of 1–2%, and differences between patient groups on the order of a few percent at most. These results contrast with conventional DCE-MRI applications where signal enhancement may be on the order of 100% or greater in tumors [1], [18] and 50% in multiple sclerosis [6].

The small changes associated with subtle BBB disorders will be significantly influenced by scanner noise, thereby requiring large sample sizes to minimize random noise and identify differences between groups, if present. The effects of noise on concentration estimation in DCE-MRI have been extensively investigated by Schabel and Parker [19], but they do not explicitly present results for the very low concentrations found in subtle BBB abnormalities, although their methods are equally valid for this situation. Other factors such as scanner drift and differences in background signal characteristics of different tissues might also contribute to observed signal differences and their influences need to be investigated. Furthermore, all of the DCE-MRI studies investigating these more subtle BBB disorders have used relatively simple analytical approaches, typically measuring signal enhancement over time in brain regions and inferring a direct relationship to BBB breakdown, i.e., assuming that greater signal enhancement equates to greater contrast agent concentration indicating a more abnormal BBB. This is a somewhat simplistic approach compared with established methodologies [6] that attempt to model the relationship between signal, contrast agent concentration and pharmacokinetics in order to quantify BBB abnormalities. In addition to providing a mechanism for estimating physiological parameters, the more complex modeling was developed to control for the effects of background tissue parameters, such as the pre-contrast longitudinal and transverse relaxation times (*T*_10_ and *T*_20_), and the longitudinal and transverse contrast agent relaxivities (*r*_1_ and *r*_2_), which affect the relationship between signal enhancement and contrast agent concentration. Analytical expressions describing the sensitivity of the signal enhancement–contrast agent concentration relationship to background tissue parameters have previously been obtained [19] and demonstrate that the relationship is nonlinear and dependent on the tissue parameters, such that the simple linear assumption is generally invalid. Tofts et al. [20] also performed an error analysis indicating how uncertainties in the experimental parameters propagate through to modeled pharmacokinetic parameters, although the results presented were based on breast carcinoma and not directly applicable to the low concentrations associated with mild BBB impairment.

In this work, the aim was to determine to what extent scanner noise, drift, intrinsic tissue properties (defined by *T*_10_, *T*_20_, *r*_1_ and *r*_2_) and imaging sequence parameters affect the interpretation of post-contrast signal enhancement in different tissues and over the range of values relevant to subtle BBB disorders. DCE-MRI data was acquired from patients with mild stroke, as part of a larger study investigating associations between stroke subtype and background BBB alterations [15]. This patient population exhibits a range of white matter lesion extent and was classified according to the degree of white matter abnormalities present. The DCE-MRI data were analyzed by conventional assessment of signal enhancement curves and modeling of contrast agent concentration. Phantom and volunteer data were obtained to assess the effects of background scanner noise and drift in different tissues. A theoretical analysis was performed to identify the effect of variations in intrinsic tissue and experimental parameters on the estimation of contrast agent concentration from signal enhancement.

## 2 Materials and methods

### 2.1 MRI Scanning

Sixty patients with mild ischemic stroke, diagnosed by an experienced stroke physician, underwent MRI. The local ethics committee approved the study and informed consent was obtained from all patients. Diagnostic MRI was performed on all patients, followed by DCE-MRI at least 1 month after the stroke to minimize any acute effect of the stroke on local BBB changes in the stroke lesion. Imaging was undertaken on a 1.5T MRI scanner (GE Signa LX, Milwaukee, WI, USA) with standard quadrature head coil. The diagnostic MRI was used to establish the recent infarct site and to classify white matter lesion extent [axial imaging: diffusion-weighted (TR/TE=9999/98.8 ms, 128×128 matrix, 240×240 mm FOV, 5 mm slice thickness, 1 mm slice gap); *T*_2_-weighted (TR/TE=6300/107 ms, 256×256 matrix, 240×180 mm FOV, 5 mm slice thickness, 1.5 mm slice gap); fluid attenuated inversion recovery (FLAIR) (TR/TE=9002/147 ms, 256×256 matrix, 240×240 mm FOV, 5 mm slice thickness, 1.5 mm slice gap); and gradient echo (*T*_2_^⁎^-weighted) (TR/TE=620/15 ms, flip angle *α*=20°, 240×180 mm FOV, 256×192 matrix, 5 mm slice thickness, 1 mm slice gap)]. DCE-MRI was performed using a 3D fast spoiled gradient echo (FSPGR) sequence (TR/TE=8.1/3.2 ms, 240×240 mm FOV, 256×256 matrix, 4 mm slice thickness). The sequence was run before contrast agent administration with flip angles of 2° and 12° to facilitate *T*_10_ measurement [21], [22], [23], [24], [25], [26], and the 12° acquisition was repeated 26 times with a temporal resolution of 69 s following an intravenous bolus injection of 40 ml gadodiamide (Omniscan, GE Healthcare, Chalfont St Giles, UK) into the antecubital vein. In order to assess scanner drift, the DCE-MRI protocol was also performed on six healthy volunteers without administration of contrast agent and on gadodiamide-doped water phantoms with *T*_10_ values representative of brain tissue and cerebrospinal fluid (CSF).

### 2.2 Image analysis

An experienced neuroradiologist examined the T_2_-weighted and FLAIR sequences from all patients in detail, classifying deep and periventricular white matter abnormalities according to the Fazekas scale (range 0 to 3) [27]. The scores for deep and periventricular abnormalities were averaged to give an overall Fazekas white matter rating and patients were dichotomized into those with overall Fazekas rating <1.5 (low) or ≥1.5 (high).

The DCE-MRI data were motion corrected by aligning all FSPGR acquisitions to the pre-contrast 12° acquisition using computational image realignment [28]. Maps of *T*_10_ were calculated voxel by voxel from the two pre-contrast acquisitions, *S*_*a*_ and *S*_*b*_, acquired with flip angles *α*_*a*_=2° and *α*_*b*_=12° using the formula adapted from Brookes et al. [22]

(1) $$\frac{1}{T_{10}}=\frac{1}{\text{TR}}\ln\left[\frac{S_{R}\sin\alpha_{b}\cos\alpha_{a}-\sin\alpha_{a}\cos\alpha_{b}}{S_{R}\sin\alpha_{b}-\sin\alpha_{a}}\right]$$

where *S*_*R*_=*S*_*a*_/*S*_*b*_. Signal enhancement (*E*_*t*_) maps were calculated voxel by voxel for each of the 26 post-contrast time points *t*, such that *E*_*t*_=(*S*_*t*_−*S*_0_)/*S*_0_, where *S*_0_ is the pre-contrast 12° acquisition. The signal enhancement represents the fractional signal increase above baseline, such that a value of 0 represents no post-contrast signal increase and a value of 1 represents a doubling of post-contrast signal. Maps of contrast agent concentration *C*_*t*_ (in millimolars) were estimated from *E*_*t*_ at each time point by voxel-by-voxel numerical solution of the formula given in Eq. (2) [29],

(2) $$E_{t}=\exp\left(-r_{2}C_{t}\text{TE}\right)\times \left[\frac{1-\exp\left(-P-Q\right)-\cos\alpha_{2}\left(\exp\left(-P\right)-\exp\left(-2P-Q\right)\right)}{1-\exp\left(-P\right)-\cos\alpha_{2}\left(\exp\left(-P-Q\right)-\exp\left(-2P-Q\right)\right)}\right]-1$$

where *P=*TR*/T*_10_ and *Q=r*_1_*C*_*t*_TR. This method makes the standard assumption that the post-contrast changes in *R*_1_ and *R*_2_^⁎^ are linearly related to *C*_*t*_ as determined by the contrast agent relaxivities *r*_1_ and *r*_2_.

The primary focus of this study was to measure the background status of the BBB, rather than alterations caused by the acute event, and so imaging was performed at least 1 month after stroke onset and measurements taken remotely from the site of the recent infarct. Multiple small circular regions of interest (ROIs) of three voxels' diameter were positioned to sample the calculated *T*_10_, *E*_*t*_ and *C*_*t*_ maps in white matter (84 ROIs), cortical gray matter (44 ROIs), deep gray matter (12 ROIs), CSF (10 ROIs) and major vessels (7 ROIs) on the pre-contrast 12° acquisition, using standard templates to ensure consistent sampling of brain regions blind to all other data including knowledge of post-contrast signal change. If necessary, the template ROI location was then adjusted slightly to avoid the recently ischemic lesion; however, ROIs were not adjusted to avoid white matter lesions. Measurements from all ROIs were combined for each subject and tissue type to produce overall mean and standard deviation values for *T*_10_, *E*_*t*_ and *C*_*t*_. The mean *E*_*t*_ (*E*_t_^ave^) and *C*_*t*_ (*C*_t_^ave^) were averaged over all post-contrast time points and along with *T*_10_ were averaged over all patients for each tissue type in each of the high- and low Fazekas-rated groups, to give overall mean and standard deviation values for each tissue in each group. A Student's *t* test was performed to look for significant differences in *T*_10_, *E*_*t*_^ave^ or *C*_*t*_^ave^ between the low- and high Fazekas-rated groups in each tissue.

The sensitivity of the FSPGR acquisition to scanner noise and drift was assessed using data acquired from volunteers and phantoms, processed in exactly the same way as the patient data. For the phantom data, ROIs were placed to cover the phantoms (cylindrical tubes of approximately 2 cm diameter and 10 cm length), and for volunteer data ROIs were placed as described above for the patient case. The contribution to the signal enhancement curves from scanner noise and drift was obtained by calculating the mean and standard deviation of *E*_*t*_ for each tissue type (or phantom) over all time points and by analyzing the slope of the signal enhancement profiles using standard linear regression analysis performed with the regression function in Microsoft Excel. These findings were then compared to the patient data.

### 2.3 Theory and simulation

Errors in the estimation of intrinsic tissue parameters (*T*_10_, *T*_20_, *r*_1_ and *r*_2_) on the calculation of contrast agent concentration have been extensively studied by Schabel and Parker [19] who derived analytical expressions for the relative bias in the concentration measurement resulting from a biased estimate of the intrinsic tissue parameters. They demonstrated that *T*_10_ produces a negative concentration bias that has the greatest influence of all the tissue parameters, *r*_1_ also results in a negative concentration bias but to a lesser degree than *T*_10_, while *r*_2_ produces a fairly negligible positive concentration bias, only becoming significant at very high concentrations. The concentration estimation is independent of *T*_20_ in the fast exchange regime and so this parameter need not be considered further.

To gain a further insight into whether the small changes in *E*_*t*_ (*E*_t_^diff^) observed between patient and control populations in subtle BBB abnormalities could result from a difference in intrinsic tissue parameters *T*_10_ or *r*_1_ rather than from a true difference in contrast agent concentration, a theoretical analysis was performed based on the data obtained in this study. Firstly, a representative *E*_*t*_ value was obtained for each tissue by calculating the mean post-contrast *E*_*t*_ (*E*_t_^ave^) across the entire 30-min imaging period from the group of patients with low overall Fazekas rating (<1.5). For each tissue, mean enhancement *E*_t_^ave^ was converted into contrast agent concentration *C*_t_^ave^, with *T*_10_ taken as the mean pre-contrast value in each tissue measured from all low Fazekas-rated patients and *r*_1_ (*r*_2_) assumed to be 4.3 (5.2) s^−1^ mM^−1^ in all tissues [30]. The variation in *T*_10_ or *r*_1_ required to produce the *E*_t_^diff^ observed between the low- and high Fazekas-rated patients in each tissue (see Table 1) was then calculated, assuming the concentration *C*_t_^ave^ remained fixed in each tissue. This procedure estimates the *T*_10_ or *r*_1_ change that would be required to cause the mean enhancement difference observed in subtle BBB breakdown due to white matter abnormalities, assuming that there is no difference in the contrast agent concentration between the two patient groups.

**Table 1:** Summary of *E*_t_^ave^, *C*_t_^ave^ and *T*_10_ measurements obtained from five tissues with patients grouped by Fazekas white matter lesion ratings

		Deep gray	Cortical gray	White matter	CSF	Blood
*E*_t_^ave^	*F*<1.5	0.064±0.033	0.074±0.027	0.015±0.011	0.167±0.118	1.463±0.408
	*F*≥1.5	0.070±0.033	0.077±0.026	0.018±0.010	0.132±0.091	1.664±0.445
	(*F*≥1.5)–(*F*<1.5)	9.15%	4.29%	15.02%	−23.68%	12.81%
*C*_t_^ave^	*F*<1.5	0.020±0.012	0.019±0.007	0.009±0.004	0.009±0.009	0.779±0.315
	*F*≥1.5	0.020±0.008	0.019±0.006	0.009±0.004	0.007±0.009	0.968±0.325
	(*F*≥1.5)–(*F*<1.5)	−3.07%	0.37%	−2.21%	−23.95%	21.62%
*T*_1_	*F*<1.5	1150±90	1259±78	787±78	5592±683	1486±431
	*F*≥1.5	1185±99	1266±84	849±74	5555±785	1346±336
	(*F*≥1.5)–(*F*<1.5)	2.97%	0.58%	7.57%	−0.66%	−9.84%

A more generic analysis of the effects of *T*_10_, *r*_1_ and *r*_2_ on measurements of contrast agent concentration can be found in Schabel and Parker [19]. This error analysis also enables calculation of the relative uncertainty in the estimation of contrast agent concentration *ɛ*^rel^ when varying experimental parameters such as SNR, flip angle *α*_*b*_ and the number of baseline images *N*_b_. The effect of varying these parameters was investigated for the relevant concentration range associated with subtle BBB disorders. The relationship was explored for *T*_10_ and *T*_20_^⁎^ parameters representative of blood, gray matter, white matter and CSF, while the effect of varying flip angle *α*_*b*_, number of post contrast measurements *N* and number of baseline measurements *N*_b_ was explored for white matter.

## 3 Results

### 3.1 Patient data

**Fig. 1:**
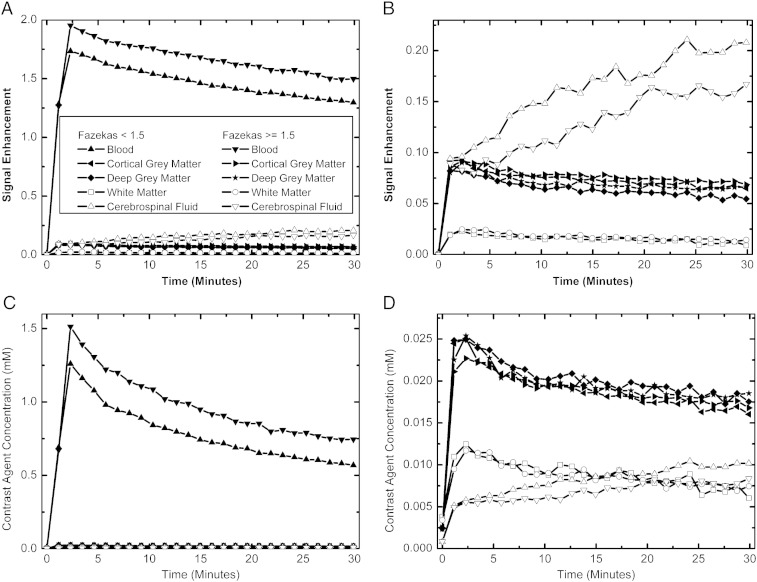
Signal enhancement (A and B) and estimated contrast agent concentration (C and D) uptake profiles obtained from blood, CSF, cortical gray matter, deep gray matter and white matter ROIs in 60 stroke patients, 32 with low overall Fazekas ratings (<1.5) and 28 with high overall Fazekas ratings (≥1.5). (A) and (C) are scaled to illustrate the blood signal relative to the other tissues, while (B) and (D) illustrate the tissue signals only. It is clear that any analysis of contrast agent uptake profiles would depend significantly on whether signal enhancement or modeled contrast agent concentration data was used.

Fig. 1 illustrates the average temporal evolution of *E*_*t*_ and *C*_*t*_ obtained from the 60 stroke patients (mean±S.D. age: 67±12 years; time from stroke onset: 68±36 days), 32 with low Fazekas rating and 28 with high rating, and Table 1 summarizes *E*_t_^ave^ and *C*_t_^ave^ measurements for each tissue. As expected, the blood signal enhances the most with *E*_t_^ave^≈1.5, which is approximately 20 times greater than either cortical gray matter (*E*_t_^ave^≈0.08) or deep gray matter (*E*_t_^ave^≈0.07). White matter enhances the least with *E*_t_^ave^≈0.02, and CSF enhances by about double that of gray matter with *E*_t_^ave^≈0.15. The relationship between tissues is noticeably altered when contrast agent concentration is considered. In this case, blood signal again has the highest estimated concentration with *C*_t_^ave^≈0.8 mM, which is roughly 40 times greater than cortical or deep gray matter which both have *C*_t_^ave^≈0.020 mM, and white matter remained the lowest of the brain tissues with *C*_t_^ave^≈0.009 mM. However, the relative difference between white and gray matter was reduced when converting from signal enhancement to contrast agent concentration. The most marked difference was in the CSF where the estimated concentration was the lowest of all tissues with *C*_t_^ave^≈0.008 mM. All tissues exhibit similar temporal trends, rising to a maximum by the second post-contrast time point and then gradually falling over time, except for CSF, which rose more progressively over time. The mean *T*_10_ values for all patients were estimated to be 1421 ms (blood), 1262 ms (cortical gray matter), 1166 ms (deep gray matter), 816 ms (white matter) and 5575 ms (CSF). The last value is significantly overestimated with the current two-flip-angle FSPGR acquisition protocol and will lead to an underestimation in the CSF concentration.

No significant differences were observed for *E*_t_^ave^ or *C*_t_^ave^ between high- and low Fazekas-rated groups in any tissues, although there was a trend towards greater *E*_t_^ave^ in the high Fazekas-rated group in brain tissues. For *T*_10_, the white matter measurement was significantly longer in the high Fazekas-rated than in the low Fazekas-rated groups (*P*=.003); a trend towards longer *T*_10_ in gray matter in the high Fazekas-rated group was observed, while both CSF and blood *T*_10_ were generally shorter in this group (*P*=ns). Therefore, in gray and white matter, these *T*_10_ differences explain the lower relative difference between patients with high and low Fazekas scores when interpreted using *C*_*t*_ data rather than using *E*_*t*_. Similarly, the differences in blood and CSF between the two groups explain the slightly greater difference observed in *C*_*t*_, rather than in *E*_*t*_.

### 3.2 Scanner noise and drift

Table 2 illustrates the mean and standard deviation of *E*_t_^ave^ for measurements obtained from phantoms with *T*_10_ values of 980 ms (brain tissue equivalent) and 2800 ms (CSF equivalent), six noncontrast volunteers (mean±S.D. age: 33±4 years) and all 60 stroke patients. Also tabulated are the slope, *R*^2^ and *P* value obtained from performing standard linear regression analysis on the data. The phantom and volunteer data indicate that scanner drift is generally well controlled on our system with a slight upward drift in signal being observed. To put these results into context, they can also be described in terms of the measured signal values. The typical signal enhancement equivalent to a change of one signal unit was measured by estimating the mean baseline signal (*S*_0_) in each tissue. The baseline signal values were 58, 52, 64, 20 and 44 for deep gray matter, cortical gray matter, white matter, CSF and blood, respectively, giving signal enhancement equivalent to one signal unit (i.e., 1/*S*_0_) of 0.017, 0.019, 0.016, 0.050 and 0.023, respectively. For brain tissue, Table 2 indicates that scanner drift and noise are well within a single signal unit in both volunteers and the phantom equivalent. For CSF, the drift was slightly greater, reaching a maximum of around 1.5 signal units after 30 min in the volunteer data and around 1 signal unit in the phantom data. For the patient data, with administered contrast agent, the mean post-contrast signal enhancement is equivalent to about 4 signal units in gray matter, 1 in white matter, 3 in CSF and 64 in blood, with changes over the imaging period following the first post-contrast time point being around −1.3 in gray matter, −0.5 in white matter, 2.2 in CSF and −15 in blood. These small signal differences will be influenced by discretization errors, as the signal is sampled as integer values. However, as the contrast agent uptake curves are obtained by averaging data from many voxels, these effects are expected to largely cancel out. Simulations performed based on the data obtained in this study indicate that the discretization error for white matter would be less than 0.01% for data averaged from 1000 voxels, far fewer than that used to generate the curves in Fig. 1. Nevertheless, if the ultimate aim is to compare data on a voxel-by-voxel basis, then discretization errors need to be reduced, possibly by improving scanner electronics or the procedure used for setting the receiver gain.

**Table 2:** Average post-contrast signal enhancement *E*_t_^ave^ and linear regression analysis results from phantoms, healthy volunteers and 60 mild stroke patients

	Tissue	*E*_*t*_^ave^	Slope	*R*^2^	*P* value
Phantom	*T*_1_=982 ms	0.001±0.004	0.00005	0.01	.61
	*T*_1_=2800 ms	0.012±0.061	0.00137	0.74	<.01
Volunteer	Deep gray matter	−0.006±0.003	0.00009	0.05	.27
	Cortical gray matter	0.001±0.004	0.00031	0.57	<.01
	White matter	−0.007±0.002	−0.00001	0.00	.86
	CSF	0.115±0.030	0.00250	0.55	<.01
	Blood	0.013±0.005	0.00045	0.54	<.01
Patient	Deep gray matter	0.076±0.026	−0.00073	0.79	<.01
	Cortical gray matter	0.067±0.033	−0.00072	0.89	<.01
	White matter	0.016±0.010	−0.00033	0.87	<.01
	CSF	0.150±0.107	0.00369	0.97	<.01
	Blood	1.557±0.433	−0.01124	0.49	<.01

### 3.3 Theory and simulation

The theoretical analysis demonstrated that to cause a greater signal enhancement for a given contrast agent concentration, either *T*_10_ or *r*_1_ must be increased. The 9.15% increase observed in deep gray matter *E*_t_^ave^ between high- and low Fazekas-rated patients would require the baseline *T*_10_ to be increased by 86 ms in the high Fazekas-rated group compared to the low Fazekas-rated group. While this is greater than the 35-ms increase observed, it is within experimental error. Similarly, the observed differences between high- and low Fazekas-rated groups in cortical gray matter, white matter, CSF and blood *E*_t_^ave^ of 4.29%, 15.02%, −23.68% and 12.81% would require *T*_10_ to differ by 43, 81, −1092 and 180 ms, respectively. The observed mean *T*_10_ differences in each of these tissues are 7, 62, −37 and −140 ms, which, while being consistently lower in magnitude than that required to cause the observed enhancement differences, are generally within experimental error of the simulated values due to the large error associated with these measurements. Similarly, if a difference in *r*_1_ between high- and low Fazekas-rated patients were to be responsible for the differences in *E*_t_^ave^, then *r*_1_ would need to be altered from its assumed value of 4.3 s^−1^ mM^−1^ by 0.43, 0.20, 0.94, −0.93 and 1.04 s^−1^ mM^−1^ in each of deep gray matter, cortical gray matter, white matter, CSF and blood, respectively. These changes are equivalent to 9.6%, 4.4%, 20.9%, −20.7% and 23.1% deviations from the assumed *r*_1_ in each of the respective tissues. These simulated data suggest that the signal enhancement differences seen in this study of 0.003 in cortical gray and white matter, 0.006 in deep gray matter and 0.035 in CSF could potentially result from differences in background tissue *T*_10_ or *r*_1_, as the predicted values are not unfeasibly large and usually lie within experimental error of the observed differences.

**Fig. 2:**
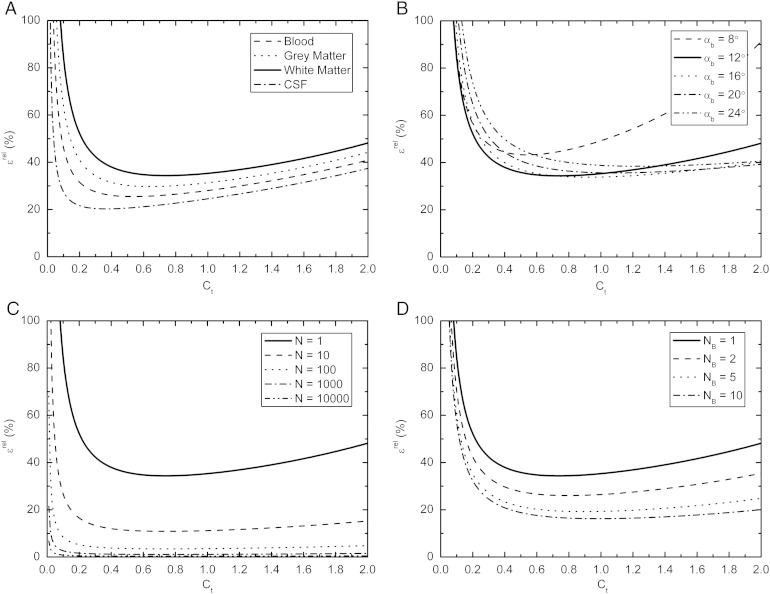
Calculated percentage error in the concentration estimation, *ɛ*^rel^ (%), as a function of the concentration, C_t_, plotted for different tissues (A); flip angles, *α*_*b*_ (B); post-contrast measurements, *N* (C); and number of baseline pre-contrast measurements, *N*_b_ (D).

Fig. 2A illustrates the relative uncertainty in the estimation of contrast agent concentration (*ɛ*^rel^) as a function of the concentration (*C*_t_) for blood, gray matter, white matter and CSF. The relationship assumes FSPGR sequence parameters as described in the MRI Scanning section with a flip angle *α*_b_=12°, a constant SNR=8 for all tissues and *T*_10_/*T*_20_^⁎^ values of 1441/290 ms for blood, 1000/49 ms for gray matter, 750/68 ms for white matter and 3000/1500 ms for CSF [19], [31]. The figure clearly demonstrates that the concentration estimation error greatly increases for concentrations typical of those measured in this study, i.e., *C*_*t*_<0.2 mM. The largest error occurs in white matter, where for typical concentrations of 0.01 mM, *ɛ*^rel^=681%. Fig. 2B demonstrates that the flip angle used in this study is well optimized for low concentration measurements in white matter, as increasing the flip angle leads to increased *ɛ*^rel^ at lower concentrations, albeit with slightly reduced error at high concentrations. Increasing the flip angle results in errors of 782% at 16°, 911% at 20° and 1053% at 24°, compared to 681% at 12° for *C*_*t*_=0.01 mM in white matter. Reducing the flip angle does slightly improve the measurement error at low concentration; a flip angle of 8° reduces the error at 0.01 mM from 681% to 663%, but at the expense of a considerably poorer performance at high concentrations. Fig. 2C demonstrates that a considerable reduction in *ɛ*^rel^ can be achieved by increasing the number of post-contrast measurements (equivalent to increasing the SNR of the experiment); however, around 10,000 measurements are required to reduce *ɛ*^rel^ to a reasonably acceptable 7%, if it is assumed that the SNR increases in proportion to √*N*. Finally, Fig. 2D demonstrates that modest reductions in *ɛ*^rel^ can also be obtained by increasing the number of baseline pre-contrast measurements, reducing *ɛ*^rel^ from 681% for *N*_b_=1, 587% for *N*_b_=2 and 500% for *N*_b_=10, provided that scanning time constraints and patient compliance allow.

## 4 Discussion

Fig. 1 and Table 1 indicate that post-contrast signal enhancement measured in mild stroke patients is small, ranging from less than 2% in white matter, 8% in gray matter, to 16% in CSF. When comparing measurements between the high- and low Fazekas-rated patients, relatively large differences were observed by imaging study standards, i.e., as high as 24% in CSF for *E*_t_^ave^ and *C*_t_^ave^, so it is somewhat disappointing that these differences did not reach statistical significance. The reason for this is due to the small absolute enhancement relative to noise, resulting in a large variance in the measurements, as illustrated in Table 1. Percentage coefficients of variation (100×S.D./mean) averaged over all tissues were 13% for *T*_1_, 49% for *E*_t_^ave^ and 56% for *C*_t_^ave^, indicating that the *T*_10_ measurement is reasonably precise, while those of *E*_t_^ave^ and *C*_t_^ave^ are considerably less so.

The theoretical error analysis demonstrated that around 10,000 measurements were required in order to reduce the concentration measurement error *ɛ*^rel^ to around 7% in white matter. In the experimental setup used in this study with 84 white matter ROIs of size nine voxels, 756 white matter voxels were measured per patient and therefore data from around 13 patients would be required to achieve a 7% error. Given that the individual voxel measurements are not independent, it is unlikely that the SNR will scale perfectly by √*N*, but these theoretical findings fit reasonably well with our empirical observation that the contrast agent uptake curves become reasonably smooth and consistent after around 20 patients, although many more patients may be required to detect very small differences. The experimental setup appears to be well optimized with regard to flip angle choice, but future studies could benefit by acquiring additional pre-contrast baseline measurements, as indicated in Fig. 2D.

Some of the variance introduced in the measurements of *E*_t_^ave^ and *C*_t_^ave^ will result from the use of a constantly administered contrast agent volume resulting in different doses being administered to different patients. The average mass of the patients was 76±15 kg (mean±S.D.), i.e., a coefficient of variation of 20%, with the average mass of the high Fazekas-rated patients being 13% lower than that of the low Fazekas-rated patients. Therefore, the more abnormal patients would have received a slightly higher contrast agent dose which appears to be reflected in the measured blood *E*_t_^ave^ and *C*_t_^ave^. Clearly, future studies should use a constant contrast agent dose for all patients if signal enhancement or contrast agent concentration curves are going to be analyzed to avoid potentially erroneous conclusions being made.

The strong influence of noise is clearly evident when comparing the patient data with measurements obtained in phantom and volunteer data with no administered contrast agent. With the exception of the blood measurements, the differences between high- and low Fazekas-rated patients (Table 1) are comparable in magnitude to the standard deviation of the measurements obtained in the phantom and volunteer data with no administered contrast agent (Table 2).

Scanner drift appears to be reasonably well controlled in all tissues except for CSF, as the post-contrast signal changes in patients are generally an order of magnitude greater than those observed in the phantom and volunteer cases. Furthermore, the small amount of drift observed in phantoms and volunteers generally opposes the trend observed in patients with contrast agent administered. However, in CSF, drift measured in phantoms and volunteers was of comparable magnitude to that observed post-contrast in patients, suggesting that scanner drift may significantly influence the enhancement profiles observed in CSF. This raises the issue of how to perform a correction because our data indicate that the degree of drift is dependent on the tissue type, suggesting that a simple correction based on a single phantom may not be sufficient. Furthermore, the drift itself is small, so measurements will be influenced by noise and likely difficult to reliably estimate for correction of an individual patient's data set. Therefore, scanner drift may introduce tissue-dependent systematic deviations in signal enhancement profiles, which, on our system, are particularly noticeable for higher *T*_10_ values, such as those found in CSF. It is possible that CSF flow influences the in vivo measurements, but at present we do not have an explanation for the differential drift observed in phantoms.

Converting signal enhancement profiles to contrast agent concentration noticeably altered the relationships between the different tissues for both subject groups. This arises due to the difference in *T*_10_ values between tissues and the nonlinear relationship between enhancement and concentration given by Eq. (2) and clearly illustrated in Fig. 2 of Schabel and Parker [19]. These results demonstrate that it is dangerous to assume that signal enhancement consistently relates to the amount of contrast agent present in any given tissue, compared to others, when those tissues differ in their intrinsic parameters *T*_10_ or *r*_1_. This emphasizes the importance of selecting an appropriate control group, with a view to minimizing these differences. Similarly, comparing the same tissue in a normal state and differing degrees of disease will not be consistently represented by signal enhancement, if *T*_10_ or *r*_1_ is altered during the disease process. Thus, a change in *T*_10_ or *r*_1_ either as part of, or associated with, the disease process can affect the changes observed in signal enhancement. For example, in addition to increased leakage of contrast agent, a common consequence of BBB breakdown is an increase in tissue water content. This elevated water content will lead to local changes in *T*_10_ and *r*_1_ that alter the observed signal enhancement, in addition to the change resulting from increased contrast agent concentration. Previous work suggests that *T*_10_ would be elevated in tissue with greater water content, while *r*_1_ is related to tissue solids content and reduces in tissue with greater water content [32], [33]. The enhancement–concentration relationship defined by Eq. (2) indicates that these would produce opposing effects, with increased *T*_10_ leading to greater signal enhancement and reduced *r*_1_ leading to lower signal enhancement in tissue with greater water content. Therefore, when signal enhancement is interpreted, it is not possible to know whether enhancement differences are due to a true difference in contrast agent concentration or to differences in *T*_10_ and/or *r*_1_. Using a model, such as that proposed in Eq. (2), to calculate contrast agent concentration attempts to overcome these limitations, provided that *T*_10_ and *r*_1_ can be reliably estimated for all tissues.

Unfortunately, it is not straightforward to measure either of the tissue parameters, *T*_10_ or *r*_1_, in the clinical setting with a high degree of reliability over the full range of values found in the brain. For example, while providing a relatively fast measurement, the two flip angle *T*_10_ measurement procedure used in this work overestimated *T*_10_ at greater values, most notably in CSF. This overestimation only results in a modest underestimation of *C*_*t*_, but if accurate CSF measurements are required, the *T*_1_ measurement procedure should be improved, while still maintaining a clinically acceptable imaging time. Reliable estimation of *r*_1_ is even more challenging, and a significant weakness of current DCE-MRI methodologies is the reliance on an assumed in vitro value for the *r*_1_ relaxivity. This is despite relaxivity measurements being known to vary significantly between (ex vivo) tissue samples measured thus far, although at least the relaxivity appears to consistently describe a linear relationship between reciprocal *T*_1_ change and contrast agent concentration at all but the most extreme concentrations [33], [34], [35], [36], [37]. However, as a feasible method for direct measurement of contrast agent concentration in living human tissue remains elusive, relaxivity properties of in vivo brain tissues (whether normal or diseased) remain largely unknown.

While the influence of *T*_10_ and *r*_1_ on the interpretation of signal enhancement curves is potentially significant, their effects are frequently ignored, particularly in the case of *r*_1_. This has been accepted in the community because traditional applications of DCE-MRI in tumors and MS produce very large signal enhancement, compared to normal tissues or subtle BBB disorders. Therefore, it is likely that such changes do arise from significant contrast agent uptake rather than from *T*_10_ or *r*_1_ alterations which would have to change by unfeasibly large amounts. Furthermore, when the enhancement is so great, there is a lesser requirement to measure *T*_10_ or *r*_1_ to such a high degree of accuracy, as small errors are unlikely to alter the overall conclusion, even though more subtle differences may be lost. In contrast, for subtle BBB disorders exhibiting small enhancement differences, relatively small differences in *T*_10_ or *r*_1_ could radically alter the conclusions drawn. As a result, *T*_10_ or *r*_1_ really needs to be known with a high degree of accuracy and accounted for when interpreting DCE-MRI results in subtle BBB disorders.

This work has described the limitations of directly inferring contrast agent concentration from signal enhancement curves in the context of subtle BBB disorders. However, it should be noted that even if a reliable estimation of contrast agent concentration profiles in each tissue is obtained, it is only a first step towards obtaining a quantitative estimate of BBB disruption. Unfortunately, MR-based measurements originate from relatively large voxels containing several different tissues or cellular environments, making it difficult to separate the contribution from each biological compartment. This is particularly important for tissues with high blood volume as this can make a particularly large contribution to the estimated concentration. In practice, pharmacokinetic modeling is used to relate the contrast agent concentration in the different compartments to underlying physiological parameters. While such models have been applied to DCE-MRI data of tumors and multiple sclerosis [6], none has modeled exchange to and from the CSF, which may be necessary in more subtle disorders [14]. Statistical modeling has also been employed, but great care is required to ensure that parameters are adequately modeled between tissues. Further work is required to establish whether these complex models can be supported by the data generated from DCE-MRI studies of subtle BBB disorders. It may be that other contrast agents need to be investigated with the aim of increasing the signal enhancement compared to that from gadodiamide, or scanner electronics and gain setting improved to increase the dynamic range of signal capture and reduce the influence of noise and signal discretization error. However, if the ultimate goal is to establish whether differences in concentration profiles are truly reflective of endothelial permeability in subtle disorders, then a quantitative assessment is required and these problems need to be overcome.

## 5 Conclusion

DCE-MRI was performed on a group of mild stroke patients classified into two groups using the Fazekas white matter rating scale. No significant differences were found between patients with a high or low white matter rating, although there was a trend towards greater enhancement in patients with a higher degree of white matter abnormality. The effect of noise, scanner drift, intrinsic tissue parameters and imaging sequence parameters on the interpretation of the signal enhancement profiles was assessed. Background noise was found to be comparable in magnitude to the observed differences, while scanner drift had less influence except in the CSF where a progressive rise in signal was observed. Calculation of contrast agent concentration, correcting for systematic differences in intrinsic tissue parameters, noticeably altered the relationship between tissues when compared to signal enhancement measurements, although differences between patient groups remained insignificant. These results suggest that it may be inappropriate to draw conclusions about the amount of contrast agent present in a tissue, and hence it is likely BBB impairment, from signal enhancement data. Therefore, studies of subtle BBB abnormalities should establish the influence of noise, drift and intrinsic tissue parameters on their data before conclusions are drawn. If this is not done, systematic errors introduced by drift and intrinsic tissue parameters may be erroneously perceived as BBB differences between patients. Careful selection of a control population may help to compensate for these issues, provided that the parameters that influence signal enhancement are expected to be identical between the control and study populations. Nevertheless, it is clear that present acquisition and processing methodologies are some way off enabling a reliable quantitative assessment of subtle BBB abnormalities and further work is required to improve these.

## References

[1] Brix G., Semmler W., Port R., Schad L.R., Layer G., Lorenz W.J. (1991). Pharmacokinetic parameters in CNS Gd-DTPA enhanced MR imaging. J Comput Assist Tomogr.

[2] Parker G.J.M., Tofts P.S. (1999). Pharmacokinetic analysis of neoplasms using contrast-enhanced dynamic magnetic resonance imaging. Top Magn Reson Imag.

[3] Harrer J.U., Parker G.J.M., Haroon H.A., Buckley D.L., Embelton K., Roberts C., Baleriaux D., Jackson A. (2004). Comparative study of methods for determining vascular permeability and blood volume in human gliomas. J Magn Reson Imaging.

[4] Kermode A.G., Thompson A.J., Tofts P.S. (1990). Breakdown of the blood–brain barrier precedes symptoms and other MRI signs of new lesions in multiple sclerosis: pathogenic and clinical implications. Brain.

[5] Katz D., Taubenberger J.K., Hashimoto S.A. (1993). Correlation between magnetic resonance imaging findings and lesion development in chronic, active multiple sclerosis. Ann Neurol.

[6] Tofts P.S., Kermode A.G. (1991). Measurement of the blood–brain barrier permeability and leakage space using dynamic MR imaging: 1. Fundamental concepts. Magn Reson Med.

[7] Farrall A.J., Wardlaw J.M. (2007). Blood–brain barrier: ageing and microvascular disease — systematic review and meta analysis. Neurobiol Aging.

[8] Berger J.R., Nath A., Greenberg R.N., Andersen A.H., Greene R.A., Bognar A., Avison M.J. (2000). Cerebrovascular changes in the basal ganglia with HIV dementia. Neurology.

[9] Avison M.J., Nath A., Greene-Avison R., Schmitt F.A., Greenberg R.N., Berger J.R. (2004). Neuroimaging correlates of HIV-associated BBB compromise. J Neuroimmunol.

[10] Bronge L., Wahlund L.O. (2000). White matter lesions in dementia: an MRI study on blood–brain barrier dysfunction. Dement Geriatr Cogn Disord.

[11] Wahlund L.O., Bronge L. (2000). Contrast-enhanced MRI of white matter lesions in patients with blood–brain barrier dysfunction. Ann NY Acad Sci.

[12] Hanyu H., Asano T., Tanaka Y., Iwamoto T., Takasaki M., Abe K. (2002). Increased blood–brain barrier permeability in white matter lesions of Binswanger's disease evaluated by contrast-enhanced MRI. Dement Geriatr Cogn Disord.

[13] Starr J.M., Farrall A.J., Armitage P., McGunn B., Wardlaw J. (2009). Blood–brain barrier permeability in Alzheimer's disease: a case-control MRI study. Psychiatry Research.

[14] Starr J.M., Wardlaw J., Ferguson K., MacLullich A., Deary I.J., Marshall I. (2003). Increased blood–brain barrier permeability in type II diabetes demonstrated by gadolinium magnetic resonance imaging. J Neurol Neurosurg Psychiatry.

[15] Wardlaw J.M., Farrall A., Armitage P.A., Carpenter T., Chappell F Doubal F., Chowdhury D., Cvoro V., Dennis M.S. (2008). Changes in background blood–brain barrier integrity between lacunar and cortical ischemic stroke subtypes. Stroke.

[16] Soon D., Tozer D.J., Altmann D.R., Tofts P.S., Miller D.H. (2007). Quantification of subtle blood–brain barrier disruption in non-enhancing lesions in multiple sclerosis: a study of disease and lesion subtypes. Mult Scler.

[17] Soon D., Altmann D.R., Fernando K.T.M., Giovannoni G., Barkhof F., Polman C.H., O'Connor P., Gray B., Panzara M., Miller D.H. (2007). A study of subtle blood–brain barrier disruption in a placebo-controlled trial of natalizumab in relapsing remitting multiple sclerosis. J Neurol.

[18] Andersen C., Jensen F.T. (1998). Differences in blood–tumour-barrier leakage of human intracranial tumours: quantitative monitoring of vasogenic oedema and its response to glucocorticoid treatment. Acta Neurochir.

[19] Schabel M.C., Parker D.L. (2008). Uncertainty and bias in contrast concentration measurements using spoiled gradient echo pulse sequences. Phys Med Biol.

[20] Tofts P.S., Berkowitz B., Schnall M.D. (1995). Quantitative analysis of dynamic Gd-DTPA enhancement in breast tumors using a permeability model. Magn Reson Med.

[21] Wang H.Z., Riederer S.J., Lee J.N. (1987). Optimizing the precision in T1 relaxation estimation using limited flip angles. Magn Reson Med.

[22] Brookes J.A., Redpath T.W., Gilbert F.J., Murray A.D., Staff R.T. (1999). Accuracy of T1 measurement in dynamic contrast-enhanced breast MRI using two- and three-dimensional variable flip angle fast low-angle shot. J. Magn Reson Imaging.

[23] Deoni S.C.L., Rutt B.K., Peters T.M. (2003). Rapid combined T1 and T2 mapping using gradient recalled acquisition in the steady state. Magn Reson Med.

[24] Armitage P., Behrenbruch C., Brady M. (2003). Book of abstracts: Eleventh Annual Meeting of the International Society of Magnetic Resonance in Medicine.

[25] Cheng H.L.M., Wright G.A. (2006). Rapid high-resolution T(1) mapping by variable flip angles: accurate and precise measurements in the presence of radiofrequency field inhomogeneity. Magn Reson Med.

[26] Schabel M.C., Morrell G.R. (2009). Uncertainty in T1 mapping using the variable flip angle method with two flip angles. Phys Med Biol.

[27] Fazekas F., Kleinert R., Offenbacher H., Schmidt R., Kleinert G., Payer F., Radner H., Lechner H. (1993). Pathologic correlates of incidental MRI white matter signal hyperintensities. Neurology.

[28] Jenkinson M., Smith S. (2001). A global optimization method for robust affine registration of brain images. Med Image Anal.

[29] Armitage P., Behrenbruch C., Brady M., Moore N. (2005). Extracting and visualizing physiological parameters using dynamic contrast-enhanced magnetic resonance imaging of the breast. Med Image Anal.

[30] Rohrer M., Bauer H., Mintorovitch J., Requardt M., Weinmann H.J. (2005). Comparison of magnetic properties of MRI contrast media solutions at different magnetic field strengths. Invest Radiol.

[31] Siemonsen S., Finsterbusch J., Matschke J., Lorenzen A., Ding X.Q., Fiehler J. (2008). Age-dependent normal values of T2* and T2′ in brain parenchyma. AJNR.

[32] Stanisz G.J., Henkelman R.M. (2000). Gd-DTPA relaxivity depends on macromolecular content. Magn Reson Med.

[33] Pickup S., Wood A.K.W., Kundel H.L. (2005). Gadodiamide T1 relaxivity in brain tissue in vivo is lower than in saline. Magn Reson Med.

[34] Donahue K.M., Burstein D., Manning W.J., Gray M.L. (1994). Studies of Gd-DTPA relaxivity and proton exchange rates in tissue. Magn Reson Med.

[35] Ibrahim M.A., Emerson J.F., Cotman C.W. (1998). Magnetic resonance imaging relaxation times and gadolinium-DTPA relaxivity values in human cerebrospinal fluid. Invest Radiol.

[36] Dimicoli J.L., Patry J., Poupon J., Volk A. (2003). On the use of R1 and R2* for measurement of contrast agent concentration in isolated perfused rat liver. NMR Biomed.

[37] Shuter B., Tofts P.S., Wang S.C., Pope J.M. (1996). The relaxivity of Gd-EOB-DTPA and Gd-DTPA in liver and kidney of the Wistar rat. Magn Reson Imag.

